# Salicylic Acid Biosynthesis in Plants

**DOI:** 10.3389/fpls.2020.00338

**Published:** 2020-04-17

**Authors:** Hannes Lefevere, Lander Bauters, Godelieve Gheysen

**Affiliations:** Department of Biotechnology, Faculty of Bioscience Engineering, Ghent University, Ghent, Belgium

**Keywords:** salicylic acid biosynthesis, isochorismate synthase, phenylalanine ammonia-lyase, plant defense, pathogen infection

## Abstract

Salicylic acid (SA) is an important plant hormone that is best known for mediating host responses upon pathogen infection. Its role in plant defense activation is well established, but its biosynthesis in plants is not fully understood. SA is considered to be derived from two possible pathways; the ICS and PAL pathway, both starting from chorismate. The importance of both pathways for biosynthesis differs between plant species, rendering it hard to make generalizations about SA production that cover the entire plant kingdom. Yet, understanding SA biosynthesis is important to gain insight into how plant pathogen responses function and how pathogens can interfere with them. In this review, we have taken a closer look at how SA is synthesized and the importance of both biosynthesis pathways in different plant species.

## Introduction

Salicylic acid (SA) was reported to play a role in disease resistance in tobacco plants by White already in 1979 (White, 1979). Since then, the importance of SA in plant defense to biotic and abiotic stimuli has been well established. SA levels are known to increase in many pathosystems upon infection with viruses, fungi, insects, and bacteria (Ogawa et al., 2006; Kim and Hwang, 2014; Hao et al., 2018; Zhao et al., 2019), and exogenous SA treatment boosts the defense system of the host (Nahar et al., 2011; Wang and Liu, 2012; Tripathi et al., 2019; Zhang and Li, 2019). Plants overexpressing NahG, a salicylate hydroxylase degrading SA, are unable to accumulate SA upon pathogen infection and are impaired in their systemic acquired resistance (SAR), a broad-spectrum systemic resistance acquired after a primary infection (Lawton et al., 1995). Although SA is essential for SAR, it is probably not the mobile signal. SAR is orchestrated by a collaboration between SA and pipecolic acid (Hartmann and Zeier, 2019; Huang et al., 2020). Despite the importance of SA in plant defense, its biosynthesis is not fully understood. In this review paper, we have focused on SA biosynthesis in the dicot *Arabidopsis thaliana* and the monocot rice (*Oryza sativa)*. Still, there are also some interesting observations made of other plants that we will touch on in this paper. For more detailed information regarding transport, perception, and signaling, we refer the reader to some recent reviews (Maruri-Lopez et al., 2019; Pokotylo et al., 2019; Zhang and Li, 2019).

## SA Biosynthesis and Metabolism in Plants

**FIGURE 1:**
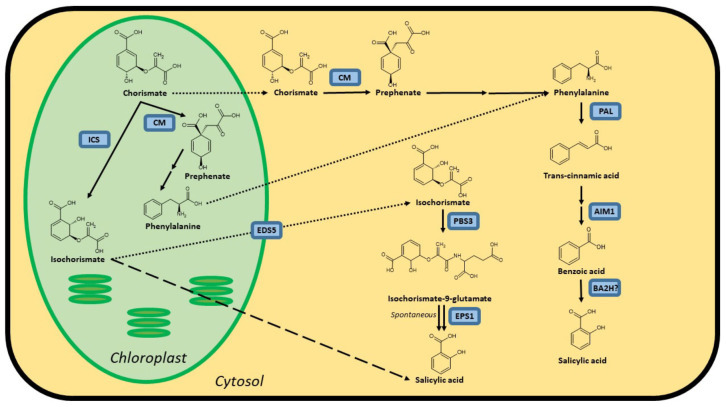
Possible biosynthesis routes for SA in plants. Full lines are conversion steps, dotted lines are transport from chloroplast to cytosol, the dashed line is an alternative, unknown biosynthesis route. The question mark indicates an unidentified protein. It is unclear whether the steps leading up to phenylalanine are performed in the chloroplast or cytosol, or in both simultaneously, as there are chloroplastic and cytosolic CMs. Enzymes are indicated in blue and are abbreviated as follows: ICS, isochorismate synthase; CM, chorismate mutase; PAL, phenylalanine ammonia-lyase; AIM1,abnormal inflorescence meristem1; BA2H, benzoic acid 2-hydroxylase; EDS5, ENHANCED DISEASE SUSCEPTIBILITY 5; PBS3, avrPphB SUSCEPTIBLE3; EPS1, ENHANCED PSEUDOMONAS SUSCEPTIBILITY 1. In Arabidopsis, *sid1* mutants are loss-of-function *eds5* mutants, while *sid2* mutants are loss-of-function *ics1* mutants.

It is widely accepted that plants possess both an isochorismate synthase (ICS) and phenylalanine ammonia-lyase (PAL) pathway to synthesize SA, both starting from chorismate (Figure 1). However, not all enzymes catalyzing these pathways have been identified in plants. The importance of these pathways for the biosynthesis of SA varies in different plant species. In *Arabidopsis*, the ICS pathway is the most important, while the PAL pathway seems to be more important for SA accumulation in rice. Both pathways contributing equally is also a possibility, as is the case in soybeans. Furthermore, SA biosynthesis regulation can even be different within the plant. In rice, for example, the basal SA levels in shoots are much higher than in roots (Silverman et al., 1995; Duan et al., 2014).

Salicylic acid can undergo several modifications in the plant. Most of them cause SA to become inactive. When SA is glucosylated, SA glucoside (SAG) can be produced. This compound can be stored in the vacuole in large quantities (Dean et al., 2003). As a result of glucosylation by Salicyloyl glucose ester (SGE) is another SA sugar conjugate that can be formed in plants. Methylation increases the membrane permeability of SA and makes it more volatile. This derivative can be released from the plant and serves as a cue for plant–insect interactions (Snoeren et al., 2010). Another major modification is amino acid (AA) conjugation, possibly involved in SA catabolism (Mackelprang et al., 2017). Hydroxylation of SA results in 2,3- and 2,5 dihydroxybenzoic acid (2,3-DHBA and 2,5 DHBA) (Dempsey et al., 2011). Recently, a glycosyltransferase has been identified that can convert MeSA to MeSA glucoside (MeSAG) (Chen et al., 2019).

## ICS Pathway in Plants

The first pathway for SA biosynthesis starts from chorismate, which is converted into isochorismate (IC) by ICS (Figure 1; Catinot et al., 2008; Abreu and Munne-Bosch, 2009; Hao et al., 2018). This pathway was first discovered in bacteria. In *Pseudomonas* species, SA is synthesized for the production of siderophore pseudomonine. The *PmsCEAB* gene cluster plays a key role in this biosynthesis (see Table 1). *PmsC* shows high sequence similarity with ICS in *E. coli*, where this enzyme catalyzes the conversion of chorismate to IC (Mercado-Blanco et al., 2001). The *PmsB* gene encodes an isochorismate pyruvate-lyase (*IPL*) gene, which converts IC to SA. This means that SA is synthesized from chorismate in a two-step process in *Pseudomonas.*

**TABLE 1:** Exemplary genes involved in the biosynthesis of salicylic acid.

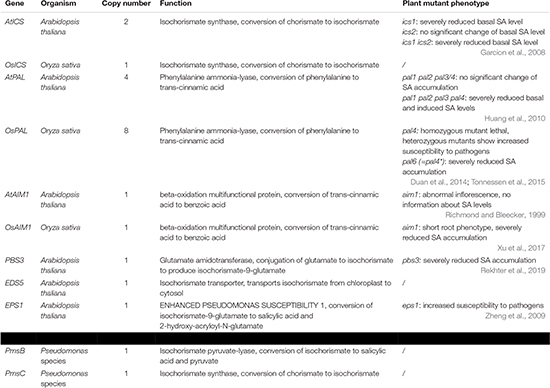

Above black separation: plant genes; below black separation: bacterial genes. *PAL6 from Duan et al. (2014) has the same locus number as *PAL4* from Tonnessen et al. (2015) (LOC_Os02g41680).

The number of ICS homologs is limited within plant genomes, with the majority of the plants having one or two gene copies. The subsequent step was, until recently, presumed to be catalyzed by IPL, but this enzyme has only been discovered in bacteria (Mercado-Blanco et al., 2001). Two 2019 studies showed that SA synthesis via ICS in *Arabidopsis* differs from that in bacteria. Amino acid conjugation of IC, followed by spontaneous decomposition or enzymatic conversion, results in the formation of SA. The gene responsible, *PBS3*, has been characterized in *Arabidopsis* but not in any other plants (Jagadeeswaran et al., 2007; Nobuta et al., 2007; Rekhter et al., 2019; Torrens-Spence et al., 2019).

The ICS pathway plays an important role in pathogen-induced SA accumulation in *Arabidopsis* (Nawrath and Metraux, 1999). The *Arabidopsis* genome contains two *ICS* homologs, *AtICS1* and *AtICS2* (Garcion et al., 2008). Garcion et al. (2008) demonstrated that SA accumulation, elicited by UV treatment, is severely impaired in *ics1* and *ics1 ics2* mutants. These mutants show a 90% decrease in UV-induced SA levels in *Arabidopsis* leaves. However, *ics2* mutants show no significant difference in basal or UV-induced SA levels compared to WT plants. This suggests that *AtICS1* is the main contributor for basal and UV-induced SA levels. Another study presents strong evidence for the importance of *ICS1* in *Arabidopsis*. When *AtICS1* is expressed in *N. benthamiana*, a much higher enzymatic activity is observed than for any other plant ICS tested, confirming the importance of ICS1 in *Arabidopsis* SA accumulation (Yokoo et al., 2018). The avrPphB SUSCEPTIBLE3 (PBS3) enzyme has recently been shown to be responsible for the conversion of IC to SA. Knock-out mutants in the *PBS3* gene show severely lowered SA and SAG levels, indicating its critical role in SA biosynthesis in *Arabidopsis.* PBS3 catalyzes the conjugation of IC and glutamate to produce isochorismate-9-glutamate. This compound can be converted by ENHANCED PSEUDOMONAS SUSCEPTIBILITY 1 (EPS1), an acyltransferase (Torrens-Spence et al., 2019), or spontaneously decompose into SA and 2-hydroxy-acryloyl-N-glutamate (Rekhter et al., 2019). However, *pbs3* knock-out mutants, as opposed to *ics1* mutants, still accumulate SA upon pathogen inoculation (Lee et al., 2007). Furthermore, *eps1* knock-out mutations do not completely prevent SA biosynthesis (Torrens-Spence et al., 2019), suggesting that IC-derived SA biosynthesis is still not fully understood and that a PBS3/EPS1 independent pathway might be present. Since ICS is located in the plastid and PBS3 in the cytoplasm, IC needs to be transported out of the plastid. The ENHANCED DISEASE SUSCEPTIBILITY 5 (EDS5) protein, a MATE transporter, is thought to be responsible for this transport, and *eds5* mutants, formerly *sid1* mutants, show much lower levels of SA upon pathogen infection (Nawrath and Metraux, 1999; Nawrath et al., 2002).

In rice, *ICS* is a single copy gene. In contrast to *Arabidopsis*, there is relatively little evidence for the importance of the OsICS enzyme in SA biosynthesis. The transcription factor OsWRKY6 has been suggested to be responsible for SA accumulation by activating the *OsICS* gene (Choi et al., 2015). However, WRKY transcription factors regulate a multitude of defense-related genes, such as *PAL*, which could also be responsible for this increase in SA (Liu et al., 2005, 2018). Furthermore, it was shown that OsICS has a very low level of enzymatic activity compared to the *Arabidopsis* homolog (Yokoo et al., 2018). In addition, there is no published research associating *OsICS* with disease resistance in rice. In conclusion, the ICS pathway might not be the main route for SA production in rice.

## PAL Pathway in Plants

While the idea of the ICS pathway as a production route for SA in plants is relatively new and based on observations in bacteria, the importance of the PAL pathway has been known for much longer. While this pathway can be responsible for the biosynthesis of SA, it should be considered that PAL is an upstream enzyme that leads to many other possibly defense-related compounds (Dixon and Paiva, 1995). Chorismate mutase (CM) is a key enzyme in the biosynthesis of SA and is responsible for catalyzing the conversion of chorismate to prephenate (Figure 1). Most plant species have several *CMs* in their genome. It is, however, more relevant to consider the conversion steps that lead from phenylalanine (Phe) to SA (Figure 1), as the steps before this metabolite branch into many other biosynthetic routes. The enzyme PAL converts Phe into trans-cinnamic acid (tCA) and has been identified in many plant species. In most cases, PAL is only able to perform this single reaction. Yet, some have a secondary tyrosine ammonia-lyase (TAL) activity (Rosler et al., 1997; Cass et al., 2015; Barros et al., 2016). *PAL* genes are present in many copies in the genome and are differentially expressed between plant tissues, which makes generalization hard to come by Reichert et al. (2009). Mutant analysis in *Arabidopsis* has identified another key player in the PAL pathway: abnormal inflorescence meristem1 (AIM1), named after the phenotype of a knock-out plant (Richmond and Bleecker, 1999). *AIM1* has been identified in *Arabidopsis* and rice and is a member of the multifunctional protein (MFP) family (Rylott et al., 2006; Arent et al., 2010). These play a major role in fatty acid metabolism and are also required for the metabolism of amino acids and hormones. *AIM1* is able to catalyze the conversion of tCA into benzoic acid (BA). It functions as a beta-oxidation enzyme and thus has many more substrates than tCA, such as fatty acids. This makes knock-out plants in this gene complex to interpret, but nonetheless valuable for studies on SA biosynthesis and defense responses. The last step, converting BA into SA, is catalyzed by a presumed benzoic acid hydroxylase. This enzyme has not yet been identified, perhaps due to the wide variety of enzymes that could fulfill this role. A study from 1995 suggested that a P450 monooxygenase is able to take on this role in tobacco (Leon et al., 1995), but further results were not published.

In *Arabidopsis*, the number of *PAL* homologs is a modest four. Single and multiple mutants for these genes were extensively studied. The generated double and triple mutants showed no difference compared to WT plants in their basal SA content nor upon infection with *Pseudomonas syringae* pv *tomato* DC3000 (*Pst* DC3000). In the quadruple mutants, basal and pathogen-induced SA levels were 25% and 50% of that of WT, respectively. The quadruple mutants were also more susceptible than WT to *Pst* DC3000 (Huang et al., 2010). However, *pal* quadruple mutants showed developmental defects. This might cause *ICS*-derived SA biosynthesis to be impaired and these mutants are therefore not ideal for studying *PAL* contribution to SA biosynthesis in *Arabidopsis* (Huang et al., 2010).

The rice *PAL* genes have been extensively studied for their role in stress responses (Giberti et al., 2012; Tonnessen et al., 2015; Li et al., 2018; Yu et al., 2018; Fang et al., 2019). Nine rice genes have been annotated as *PALs*. However, *PAL9* was later shown to possess tyrosine aminomutase activity, hence its recent renaming as TAM1 (Yan et al., 2015). Discoveries like this make it questionable whether all of the other eight genes possess PAL activity. *PAL 1-7* genes co-localize with disease resistance QTLs, indicating their role in plant defense (Tonnessen et al., 2015). Nevertheless, some *PAL* genes are more involved in rice defense than others. *Pal6* knock-out rice plants have a 70–77% decrease in PAL activity and show an increased susceptibility to *M. oryzae*, indicating the relative importance of PAL6. These *pal6* plants have a 60% decrease in SA content, despite a 3-fold upregulation of *ICS*, indicating that the PAL pathway is the main production pathway for SA in rice (Duan et al., 2014). *PAL6* of Duan et al. (2014) is the same gene as *PAL4* of Tonnessen et al. (2015). Heterozygous mutant *pal4* plants (Tonnessen et al., 2015), which show a greater than twofold drop in *PAL4* transcript level compared to WT, were more susceptible to *X. oryzae* pv. *oryzae* and to *R. solani*. They also show a highly induced *PAL2* expression, while *PAL6* expression is reduced [PAL gene numbering according to Tonnessen et al. (2015)]. These observations highlight the complex interplay between the different *PAL* genes upon pathogen infection and suggest gene-specific *PAL* induction upon infection with a distinct pathogen (Tonnessen et al., 2015).

An abnormal inflorescence meristem1 (*aim1*) mutant was identified in *Arabidopsis* (Richmond and Bleecker, 1999). *Arabidopsis* has another gene (*MFP2*) that is homologous to *AIM1*, with a similar beta-oxidation function (Richmond and Bleecker, 1999). AIM1 has been shown to be necessary in *Arabidopsis* for the production of BA, a precursor of SA (Bussell et al., 2014). This makes it plausible that AIM1 is in large part responsible for the conversion of tCA to BA, which renders it a key enzyme in the PAL pathway. Yet, up to this point, no studies on SA levels in AIM1-deficient *Arabidopsis* plants have been published. Recently, a rice plant with reduced root meristem was identified with a mutation in the ABNORMAL INFLORESCENCE MERISTEM 1 (*AIM1*) gene (Xu et al., 2017). Rice panicles remained relatively normal, suggesting *AIM1* has a different role than in *Arabidopsis*. *Aim1* rice mutants only have a 30% of the SA levels in roots, compared to WT plants. Furthermore, they show a 2-fold decrease in BA content and a 6-fold increase in t-coumaric acid (tCA), a precursor to BA. This indicates that AIM1 takes part in catalyzing the conversion of tCA to BA in rice. This makes it an interesting target for future research. Unfortunately, infection assays have not yet been performed on *aim1* rice plants. It should be noted that there are three more members in the rice *MFP* family, which could partly contribute to the conversion of tCA to BA.

Plant SA biosynthesis pathway preference has recently been reviewed by Hartmann and Zeier (2019). The few other studied plant species also show dominance of either the ICS or the PAL pathway for their SA production (Coquoz et al., 1998; Ogawa et al., 2006; Chang et al., 2008; Hao et al., 2018). Nevertheless, soybean (*Glycine max*), which has two *ICS* and five *PAL* homologs, shows equally important roles for the ICS and PAL pathway in its SA accumulation. When infected with *P. syringae* pv. g*lycinea* (*Psg*) or *Phytophthora sojae*, a threefold increase in SA is observed. Silencing of either the *PAL* or the *ICS* pathway resulted in significantly reduced levels of SA accumulation upon pathogen infection. Furthermore, these silenced plants were more susceptible to infection by either of these pathogens (Shine et al., 2016).

## Pathogens Interfering With SA Biosynthesis

Several pathogen effectors have been identified affecting SA levels and signaling. *P. sojae* and *Verticillium dahliae* secrete isochorismatases when infecting *Arabidopsis* and cotton (*Gossypium hirsutum L.*). Isochorismatase converts IC to 2,3-dihydro-2,3-dihydroxybenzoate (DDHB), depleting IC as SA precursor, and consequently decreasing SA production. These effectors are necessary for full pathogenesis in the plant (Liu et al., 2014). Several plant-parasitic nematode species also produce isochorismatases (Bauters et al., 2014). Plant-parasitic nematodes also secrete CM (Haegeman et al., 2013), that can divert chorismate away from the ICS pathway, thus limiting SA accumulation (Wang et al., 2018). In maize, the fungus *Ustilago maydis* also secretes CM (Djamei et al., 2011). This enzyme has been identified in various plant pathogens, and loss-of-function mutants show a decreased pathogenicity. Both CM and ICM divert metabolites away from the ICS pathway, which could be an indication for its general importance upon pathogen infection. A different approach is the breakdown of SA in the plant. *U. maydis* can express a salicylate hydroxylase (Shy1), which is not secreted. Although this enzyme degrades SA, it has not yet been linked with virulence (Rabe et al., 2013). When a bacterial *NahG* gene is expressed in tobacco, it has been shown to effectively degrade SA (Gaffney et al., 1993). *Fusarium graminearum* also produces a salicylate hydroxylase (*FgNahG*), and wheat plants infected with a *fgnahg* mutant strain showed fewer disease symptoms and a higher level of SA accumulation in wheat spikes compared to infection with WT *F. graminearum* (Qi et al., 2019). In addition, pathogens do not only interfere with SA accumulation but can also disrupt SA signaling pathways (Tanaka et al., 2015).

## Discussion

Publications often generalize that the ICS pathway is responsible for basal and pathogen-induced SA accumulation in plants, and this is based on data from *Arabidopsis* (Djamei et al., 2011; Choi et al., 2015; Qi et al., 2018). While this seems to hold true, at least in part, for Arabidopsis, a general answer for all plant species does not seem that clear-cut. Some plants seem to mainly use the PAL pathway instead of the ICS pathway for SA biosynthesis. Unraveling the details of the pathway becomes challenging because some of the enzymes involved remain unidentified to this day, and different plants seems to have evolved in different directions. Furthermore, the high copy number of the *PAL* and *CM* genes in the genome renders these hard to study, and inactivation of one gene can influence activity of the others.

The last step in the PAL pathway is thought to be catalyzed by a benzoic acid-2-hydroxylase (BA2H). Yet, its existence has only been indirectly shown (Sawada et al., 2006). It is still possible that the conversion from Phe to SA also happens via an alternative route, as shown by several isotope feeding studies (Klämbt, 1962; El-Basyouni et al., 1964; Chadha and Brown, 1974).

While the *PBS3* gene has been identified in *Arabidopsis*, it has not been described in any other plant species. As it was reported that barley *ics* mutants do not show a difference in SA levels, we could speculate that plants that predominantly use the PAL pathway for SA biosynthesis do not contain an active *PBS3* gene and solely use *ICS* for the biosynthesis of other metabolites (Qin et al., 2019).

## Author Contributions

HL and GG designed the content of the manuscript. HL wrote the manuscript. GG and LB revised and corrected the manuscript. All authors read and approved the final manuscript.

## Conflict of Interest

The authors declare that the research was conducted in the absence of any commercial or financial relationships that could be construed as a potential conflict of interest.
