# Correction: Salicylic acid biosynthesis in plants

**DOI:** 10.3389/fpls.2025.1697849

**Published:** 2025-10-06

**Authors:** Hannes Lefevere, Lander Bauters, Godelieve Gheysen

**Affiliations:** Department of Biotechnology, Faculty of Bioscience Engineering, Ghent University, Ghent, Belgium

**Keywords:** salicylic acid biosynthesis, isochorismate synthase, phenylalanine ammonia-lyase, plant defense, pathogen infection

There was a mistake in **Figure 1** as published. Some chemical formulas were not correct. The corrected **Figure 1** appears below.

**Figure 1:**
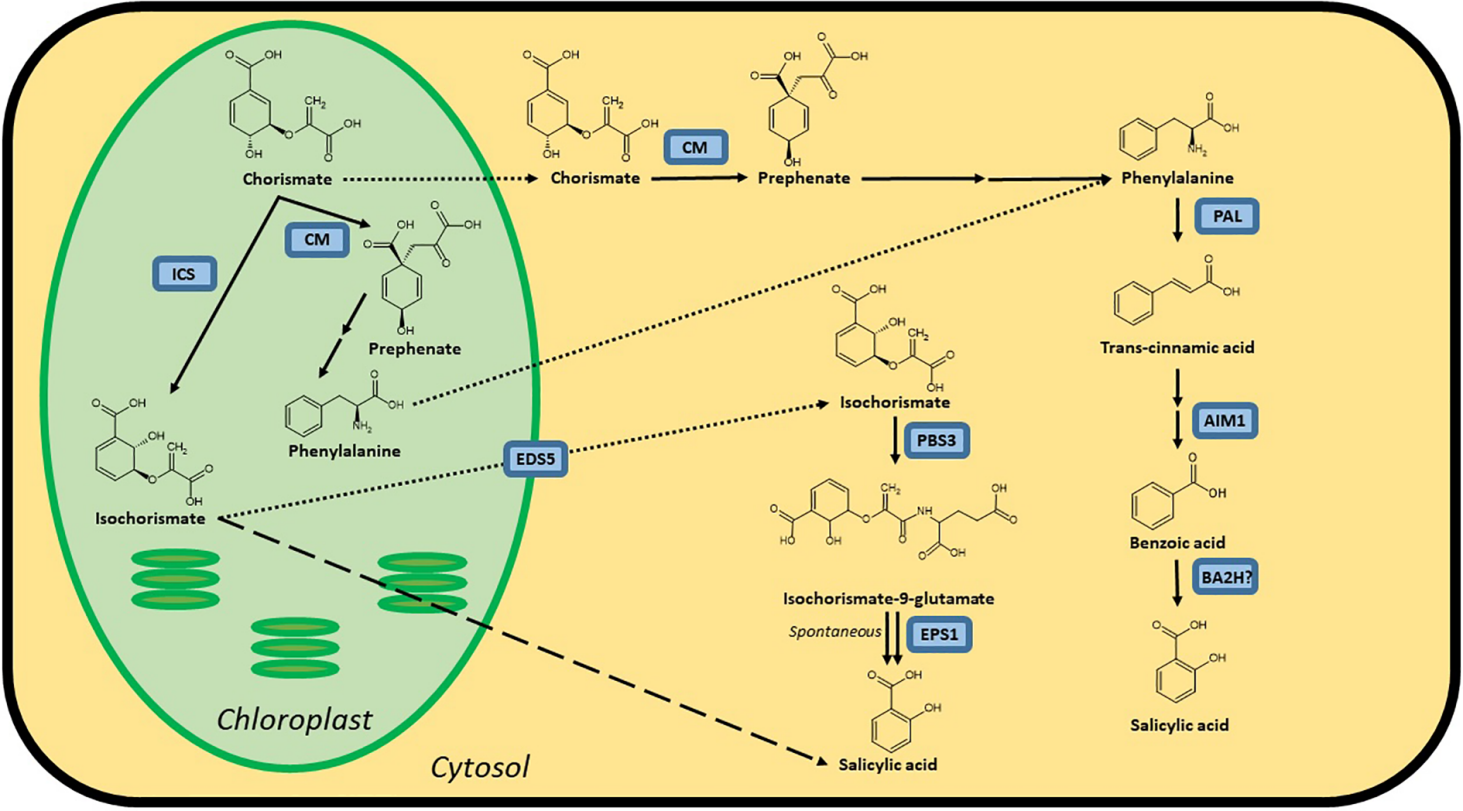
Possible biosynthesis routes for SA in plants. Full lines are conversion steps, dotted lines are transport from chloroplast to cytosol, the dashed line is an alternative, unknown biosynthesis route. The question mark indicates an unidentified protein. It is unclear whether the steps leading up to phenylalanine are performed in the chloroplast or cytosol, or in both simultaneously, as there are chloroplastic and cytosolic CMs. Proteins are indicated in blue and are abbreviated as follows: the enzymes ICS, isochorismate synthase; CM, chorismate mutase; PAL, phenylalanine ammonia-lyase; AIM1,abnormal inflorescence meristem1; BA2H, benzoic acid 2-hydroxylase; PBS3, avrPphB SUSCEPTIBLE3; EPS1, ENHANCED PSEUDOMONAS SUSCEPTIBILITY 1 and the transporter EDS5, ENHANCED DISEASE SUSCEPTIBILITY 5. In Arabidopsis, *sid1* mutants are loss-of-function *eds5* mutants, while *sid2* mutants are loss-of-function *ics1* mutants.

The original version of this article has been updated.

## Publisher‘s note

All claims expressed in this article are solely those of the authors and do not necessarily represent those of their affiliated organizations, or those of the publisher, the editors and the reviewers. Any product that may be evaluated in this article, or claim that may be made by its manufacturer, is not guaranteed or endorsed by the publisher.

